# Drivers of resource allocation for breeding under variable environments in a bet hedger

**DOI:** 10.1002/ece3.10485

**Published:** 2023-09-06

**Authors:** Daniel Oro, Cassidy Waldrep, Albert Bertolero, Meritxell Genovart

**Affiliations:** ^1^ Centre d'Estudis Avançats de Blanes – CEAB (CSIC) Blanes Spain; ^2^ Department of Biology Miami University Oxford Ohio USA; ^3^ Department of Biology University of Saskatchewan Saskatoon Canada; ^4^ Associació Ornitològica Picampall de les Terres de l'Ebre, La Galera Amposta Spain

**Keywords:** age, bet‐hedging, clutch size, food, intra‐clutch asymmetries, long‐lived bird

## Abstract

The evolutionary theory of life histories predicts that there is a trade‐off between survival and reproduction: since adult survival in long‐lived organisms is high, then breeding investment is more variable and more dependent on conditions (e.g. food availability and individual experience). Clutch features influence fitness prospects, but how a bet hedger builds its clutch in temporally varying environments is quite unknown. Using 27‐year data on 2847 clutches of known‐age breeders, we analyse how Audouin's gulls (*Larus audouinii*), a species showing a combination of conservative and adaptive bet‐hedging breeding strategies, can allocate energy by laying clutches and eggs of different sizes. Results show that both food availability and age influenced clutch size and total egg volume in a clutch. Interestingly, we found an interaction between food and age on egg parameters: total volume in two‐egg clutches, laid mostly by younger breeders, did not significantly change with food availability and the quadratic pattern in clutch size over the range of ages was less marked as long as food conditions became harsher. With increased food, females invested more by building larger first eggs, whereas they were more conservative on second and third eggs. Furthermore, asymmetries in egg volume within three‐egg clutches increased with food availability for old females. Egg size profiles of two‐egg clutches suggest that gulls should exhibit progressive reduction of the size of the third egg before shifting to a two‐egg clutch size. Food availability influenced all parameters studied, whereas age affected the amount of energy allocated for producing eggs (their size and number) but not the way of allocating those energies (i.e. asymmetries within the clutch). Despite the range of factors affecting the clutch, results suggest that females can allocate the amount of resources in a clutch optimally to increase their fitness under variable environments via bet‐hedging.

## Graphical abstract

In this study, we analysed data on the breeding investment of Audouin's gulls over a 30‐year period to understand how these long‐lived bet‐hedging birds allocate energy for breeding. We found that food availability and age influenced clutch size and the total volume of eggs in a clutch, with gulls laying more and larger eggs in years with favourable conditions and as they aged. We also found that females invested more in the first eggs in a clutch by building larger eggs with increased amounts of food, while being more conservative with the second and third eggs. The results suggest that females can allocate resources in a way that increases their fitness under variable environments through a bet‐hedging strategy.

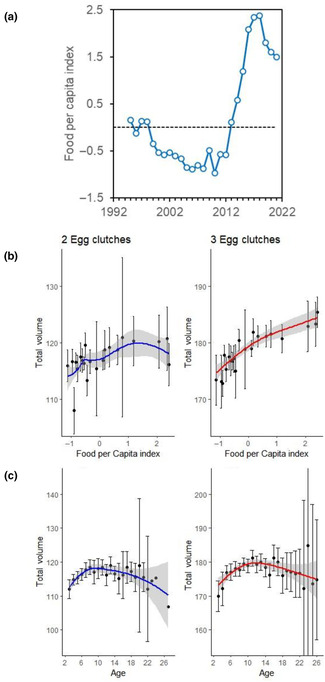

## 1 INTRODUCTION

Egg production in birds is currently recognized as an expensive process and there is increasing evidence suggesting that energy investment in terms of number and size of eggs can affect parental lifetime fitness (see reviews in Krist, 2011; Monaghan & Nager, 1997; Sockman et al., 2006; Williams, 1994). Therefore, considerable effort has been made to identify factors underlying the variability of clutch and egg size in birds. Among these factors, food availability has been reported as one of the most influential, at both inter‐ and intra‐clutch levels (Bolton et al., 1992; Clifford & Anderson, 2001; Mills, 1979; Winkler, 1985). Nevertheless, the effects of food availability on clutch and egg size are difficult to detect because of the confounding effects of several other factors (Godfray et al., 1991). Studies have identified such factors as developmental mode, for example altricial and precocial (Deeming, 2002; Starck & Ricklefs, 1998), clutch features, for example lay order (Oro et al., 1996), parental investment, for example trade‐offs between current reproduction and future reproduction (Stearns, 1992; Williams, 1966), parental quality, for example adult size, age, experience, phenotypic quality or physical condition (Bolton, 1991; Croxall et al., 1992; Davis, 1975; Murphy, 2000; Oro et al., 2014; Ortego et al., 2007), environmental condition factors, for example density dependence (Real et al., 2017), latitude (Monaghan & Nager, 1997), temperature (Heming & Marini, 2015; Nager & Van Noordwijk, 1992; Robertson, 1995), vegetation cover (Becker & Erdelen, 1986; Martinez, 2012), predation risk (Dillon & Conway, 2018) and factors related to population conditions, for example nest density (Coulson et al., 1982; Dillon & Conway, 2018; Loucif et al., 2021).

Depending on stochastic environments, the number, size and lay order of eggs laid allows female birds to allocate energetic resources differently every breeding season to optimize fitness (Brockelman, 1975), similarly to the number of different seeds a plant produces, a process that has been termed ‘multiplicity in unity’ (Herrera, 2009). An evolutionary strategy to cope with environmental variability is bet‐hedging, by which individuals would reduce variance in fitness over time resulting in a better average performance (Starrfelt & Kokko, 2012; Stearns, 1992). Since the conditions in which an organism lives can vary greatly over time, producing a range of offspring with different traits increases the chances that at least some of them will be well‐suited to the current conditions. It is known that organisms incur a trade‐off between survival and reproduction ensuing from the costs of reproduction (e.g. Stearns, 1992). According to the bet‐hedging strategy applied to birds, a conservative mean clutch size would be the most productive strategy over multiple reproductive seasons. This strategy balances the trade‐off between investing heavily in one clutch and risking reproductive failure in unpredictable conditions. It increases an individual's overall fitness by ensuring some offspring survive even in challenging environments (Starrfelt & Kokko, 2012).

Gulls are a good model to study bet‐hedging on since they have a slow life history (i.e. have a high adult survival, low recruitment, small clutches, delayed maturation and slow development and growth). Many gull species have a modal clutch size of three eggs, and they show a typical intra‐clutch pattern characterized by the relatively small size of the third egg (Kilpi et al., 1996; Oro et al., 1996; Reid, 1987b). This pattern has been interpreted in some studies to be caused by ultimate factors (i.e. as adaptive) through the brood reduction hypothesis (Slagsvold et al., 1984; Williams et al., 1993), or by proximate factors (i.e. as non‐adaptive) through the energetic, nutritional, or physiological constraints incurred by the female during clutch formation (Arnold, 1991; Jover et al., 1993; Salzer & Larkin, 1990). Gulls are rather income breeders, that is the clutch is formed using the energetic contents of the food ingested the days before laying in contrast to capital breeders that use energy stored the winter before (Bolton et al., 1993). Besides, gulls have evolved for a life history favouring constant and high survival and that they will not jeopardize that survival by laying more eggs and incurring high costs of reproduction, independently of environmental conditions in each breeding season (Monaghan & Nager, 1997).

Physiological constraints and the evolution of life histories sets a range in clutch size and asymmetries in egg volume within the clutch in long‐lived birds such as gulls. Nevertheless, there is variability in those parameters over the breeding season and individual lifetimes (Monaghan & Nager, 1997; Oro et al., 1996; Real et al., 2017). Little is known about the factors affecting how females allocate resources for each egg in the clutch under variable environments and individual conditions, such as age or experience. This is relevant for individual fitness, since larger eggs produce heavier chicks, which are more likely to be recruited into the breeding population in the coming years (Amat et al., 2001; Payo‐Payo et al., 2016). However, environmental conditions related to food may also vary within the breeding season and may decrease parental care, the development rate of chicks and the association between egg and clutch sizes and breeding success. This would all deter breeders of long‐lived species to invest large amounts of energy in the clutch (Oro, 1996; Oro et al., 1995, 1996).

Here, we analyse the effects of food availability per capita (i.e. considering density dependence) and age of the breeder on the breeding investment of Audouin's gulls (number, size and order of laid eggs) over 27 years of variable environmental conditions, including years with severe food shortage, when trade‐offs between survival and fertility would be the largest (Stearns, 1992). The challenges imposed by the long‐term monitoring of food conditions and reproductive performance on known‐age birds explain the low number of studies testing the interactions between food and age (Oro et al., 2014; Pardo et al., 2012). We tested the hypothesis that females would allocate resources preferentially to eggs with the greatest survival potential. Ruiz et al. (2000) built a bio‐energetic model to explore the costs of laying three‐egg clutches in gulls (i.e. the modal clutch size) considering the temporal dynamics of rapid yolk development (RYD). RYD is a crucial phase in the reproductive cycle of birds, during which the yolk is rapidly deposited in the developing eggs to provide the necessary nutrients for embryonic growth and development. The model predicted that significant increases of the durations of the RYD periods of second and third eggs or even significant reductions in yolk size of these eggs, might operate simultaneously to match the energy demands during clutch formation to the prevailing food conditions (Ruiz et al., 2000). This model also predicts that the volume of the first laid eggs should be clearly affected by food availability, whereas the volume of the last laid egg should not be affected by this factor (Ruiz et al., 2000). Obviously, if this is true, then all the hypotheses relying on food constraints to explain the relative size of last eggs in gulls can be ruled out, including the behaviourally mediated courtship feeding mechanism (Helfenstein et al., 2003; Salzer & Larkin, 1990) and the inverse relationship between intra‐clutch asymmetry and food availability (Kilpi et al., 1996). For assessing the prediction of the RYD model, we studied the following parameters of the clutch: total volume (as a proxy of total investment in a breeding attempt), clutch size, egg volume depending on order within the clutch, and four different indexes for exploring intra‐clutch egg size profile (see Section 2 below). Furthermore, we include the analysis of inter‐ and intra‐clutch egg size variation in two‐egg clutches in order to assess the idea that food shortages shift three‐ to two‐egg clutches because of the lack of the third egg, that is whether the two‐egg clutch pattern corresponds to first and second laid eggs of three‐egg clutches or not (Kilpi et al., 1996; Parsons, 1976). While the positive effect of age (often following a quadratic effect) on clutch and egg size and has been studied in several species of long‐lived birds (Blas et al., 2009; McCleery et al., 2008; Oro et al., 2014), little is known about the effects of age on the profile asymmetry within clutches in bet‐hedgers. Following results from a few study cases, we test the hypothesis that age, contrarily to food conditions, does not influence the asymmetry profiles of egg size within clutches (e.g. Catry & Furness, 1997; Polito et al., 2010). Finally, we tested the hypothesis that in bet‐hedgers, improved food conditions would lessen the quadratic age pattern found for breeding parameters in long‐lived species.

## 2 MATERIALS AND METHODS

### 2.1 Study area and biological model

The Punta de la Banya (40°40′ N, 0°45′ E; NW Mediterranean) has held a colony of Audouin's gulls since its colonization in 1981. The patch is a protected peninsula formed by 2500 ha of salt marshes and saltpans. Gulls are long‐lived (mean life expectancy ca. 13 years, longevity records of 33 years old, own data) and they breed seasonally. Males weigh 670 g on average (SD = 51, range = 545–755, *N* = 34), whereas females are ca. 16% lighter (mean = 560 g, SD = 49, range = 475–655, *N* = 25; Genovart, Oro, & Bonhomme, 2003). In the days before laying, males perform courtship feeding for the females and both parents contribute equally to parental care (Oro, 1998). Before eggs are laid, the process of egg formation involves several stages of growth and development. Clutch formation starts 9 days before laying the first egg and average‐sized eggs are laid in intervals of 2 days according to their position in the laying sequence (Ruiz et al., 2000). Females normally lay clutches with a mode value of three eggs (range: 1–5) but in years with exceptionally low availability of food during egg formation, females tend to have a mode value of two eggs (modal clutch size = 2; Oro et al., 1999). Hatching success tends to be high (mean percentage = 88, range = 79–93, *N* = 27 years and 7516 eggs, see below; Genovart, Oro, Ruiz, et al., 2003; Oro et al., 1996, 2014). Hatching of all eggs is relatively synchronous (all eggs hatch within 2 days), the first and second eggs both in three‐ and two‐egg clutches within the same day due to the female incubating only after the second egg is laid. Chicks do not form crèches and are partially nidifugous (they are able to leave the nest but still rely on their parents for food). They compete for food brought by the adults and their survival is partially influenced by laying order and size (last eggs being often smaller and the third hatched chick has a lower probability of surviving to fledging; Oro et al., 1996). Starvation is the main source of chick mortality (Genovart, Jover, Ruiz, & Oro, 2003; Oro et al., 1996), and mean fledging success is 0.50 chicks per pair (range = 0.01–1.07, *N* = 30 years; Genovart et al., 2018). We analyse two and three egg clutches here, which are the sizes of 90% of the clutches (*N* = 24,449) over the past 30 years of monitoring (1992–2021).

### 2.2 Egg and clutch size sampling

During each breeding season throughout 1995–2021, we measured eggs in nests with clutches of two and three eggs just before hatching. We assumed that we were monitoring first clutches only, since replacement clutches are rare in this species (Oro, 1998). Eggs were measured (length and maximum width) to the nearest 0.1 mm using a digital calliper. Egg size was estimated by internal egg volume (in cm^3^) as predicted by the equation: *V*_*i*_ = 0.000467 × length × breadth^2^ (Ruiz, Jover, & Pedrocchi, 1998).

### 2.3 Food availability

Although Audouin's gulls are considered a feeding specialist in catching clupeoids at night (Oro, 1998), they exploit discards from trawling fishery activities (70% in biomass), which are very predictable in space and time (Mañosa et al., 2007; Oro et al., 1997; Pedrocchi et al., 2002). We analysed the local industrial fishery's landing statistics from the four closest harbours within a 60 km radius of the colony. These data, collected during March and April, coinciding with the pre‐laying and laying periods, served as a proxy for yearly variability in food availability throughout the study. Previous studies show that there is a correlation between landing and the amounts of discarded fish (see details in Genovart et al., 2022; Oro & Ruiz, 1997; Payo‐Payo et al., 2016). We divided the landings by the number of breeding females each year (as well as with the number of females of the competing yellow‐legged gulls at the patch) to obtain an index of food availability per capita as a measure of the strength of density dependence (i.e. both intra‐ and inter‐specific competition), which is known to occur in the study species (Genovart et al., 2018; González‐Solis et al., 1997; Payo‐Payo et al., 2016; Ruiz, González‐Solıs, et al., 1998).

### 2.4 Age of breeders

Since 1988 to the present day, we have marked chicks each breeding season at the colony using a plastic ring with an individual alphanumeric code. Since 1995, we resighted breeding birds (either laying or incubating eggs) that were previously marked as chicks using telescopes from the distance. We assessed that individuals may start to breed at 3 years old. Owing that our dataset with marked breeding birds has encompassed the period from 1995 to 2021, we resighted 1909 breeders marked as chicks during 1988–2018 (*N* = 34,726 chicks). Since Audouin's gulls are ground‐nesting birds and they change nest location every breeding season, most resighted breeders (1569 individuals, 82% from total) were monitored only once, and the rest more than 1 year (range = 2–6). The age of monitored breeders ranged from 3 years (the first age of breeding) to 27 years old. Once a marked gull showed breeding behaviour related to egg laying, the nest was marked with a numbered plastic tag, and each newly laid egg was numbered with indelible ink according to its laying position. We measured 7516 eggs corresponding to 2847 clutches (33% and 67% of two‐ and three‐egg clutches respectively; annual mean = 95 clutches, median = 99, range = 28–173, *N* = 27 years). To ensure that actual clutch sizes were recorded, we continued to survey marked nests until no new eggs were laid for five consecutive days. Laying order was ascertained for nests detected having laid the first egg of the clutch. For three‐egg clutches in which order could not be recorded, we assigned the third laid egg as the one that hatched the later (normally 1 day later than the other two, which are very synchronous; Oro et al., 2014). This was feasible for clutches in which all eggs hatched.

Since in most cases we only resighted one bird of the couple of the monitored nest, we used the pairs in which the two adults were resighted to assess the assortative mating by age (*N* = 86). Data showed that there is a strong assortative mating (difference of age: mode = 0 years, median = 1 year, mean = 1.8 years, range = 0–10, correlation coefficient *r* = 0.69), so we assumed that the age of the adult resighted matched with the age of its breeding mate. For cases in which the two adults were marked and had different ages, we considered the integer of the mean value of the two ages.

Clutches with known‐age breeders and with known laying order were considered for the analysis of the effects of food availability and age on asymmetries and profiles of egg volume within the clutch (see below).

### 2.5 Dependent variables related to breeding investment

We analysed several metrics of breeding investment associated with the production of eggs every breeding season. We tested whether food and age influenced total volume in a clutch, clutch size (only considering clutches of two and three eggs), and egg volume depending on egg laying order.

For exploring intra‐clutch egg size profile, we used four different indexes. We first calculated an index $\beta_{i}$ of how small the last egg is compared with the arithmetic mean of the precedent eggs in a clutch i:

$$\beta_{i}=\frac{v_{j}}{\bar{\left(v_{j-2}+v_{j-1}\right)}}$$

where $v_{j}$ is the volume of the last egg in a clutch with size j [range: 2–3]. Note that for two‐egg clutches $v_{0}=0$ (i.e. there is only one value, $v_{1}$, in the denominator).

Since we are interested in the influence of food availability and age on any measure of within clutch asymmetry, we assessed not only the $\beta_{i}$ index, but also other three ratios that have been identified that may be relevant for egg laying investment (Coulson et al., 1982; Ruiz et al., 2000; Sydeman & Emslie, 1992):

$$\beta_{i}^{{\left(a:b\right)}_{j}}=\frac{v_{1}}{v_{2}}$$

$$\beta_{i}^{a:c}=\frac{v_{1}}{v_{3}}$$

$$\beta_{i}^{b:c}=\frac{v_{2}}{v_{3}}$$

where *a*, *b* and *c* represented the first, second and third eggs in a clutch; thus, $\beta_{i}^{a:c}$ and $\beta_{i}^{b:c}$ can be calculated only for three‐egg clutches, while $\beta_{i}^{{\left(a:b\right)}_{j}}$ can be calculated for each clutch size j.

### 2.6 Data analysis

Distributional assumptions and homoscedasticity were routinely checked by plotting histograms of residuals and Shapiro–Wilk normality tests (package MASS in *R*‐software) and statistical departures from normality assumptions were not detected (results not shown). Our regression models included two independent variables: age and food availability per capita. Our procedure was first to model the best fit of age for each egg parameter (i.e. total volume, clutch size, egg volume depending on laying order, *β* profiles). For this, we built regression models with different shapes of age (linear, quadratic, log). Quadratic and log shapes were tested by transforming age values (quadratic‐ and log‐transformed respectively). GLMM and GLM models (linear regression models with and without random effects respectively) were used to build those models. Models of age shape for total volume included the clutch size as a random effect, whereas models for the other dependent variables did not include random effects. Finally, we utilized the best fit of age depending on the models above for testing the effects of food availability.

GLM and GLMM models were also used to test for the effects of age and food availability (and their interaction) on total volume, clutch size and $\beta_{i}$ profiles. For models of total volume, we included clutch size in all models, since there must be a strong association (i.e. the more eggs in a clutch, the larger the total egg volume). What we were interested in those models was the potential effects of the interaction between factors (age and food availability) and clutch size, owing that the total energy invested by breeding adults was different when laying two and three eggs. We also used models of $\beta_{i}$ profiles to test whether the two‐egg clutches pattern corresponded to first and second laid eggs of three egg clutches or not. Since very few nests with the same adult were recorded over the study (3%), we did not include the adult identity as a random effect. When testing the effects of age, food availability and egg order on egg volume within clutches of three eggs, we used GLMM models. In those models, the nest was included as a random effect, since an outstanding female effect in egg size variability within the clutch is commonly found in birds, also in the study species (Lessells et al., 1989; Oro et al., 1996; Väisänen et al., 1972; Van Noordwijk et al., 1981). All models were run using *R* packages *glm* and *lmer* (for linear GLM and linear mixed effects GLMM models respectively). For GLMM models, we used the Satterthwaite's method for computing the degrees of freedom and *t*‐statistics for each factor tested.

We used the Akaike Information Criterion corrected for small sample sizes (AICc) for selecting the best model explaining our data and the weight of each model (Wt_*i*_) for the total set of models tested for each clutch parameter. Note that the Akaike weight (Wt_*i*_) is a value between 0 and 1, with the sum of weights of all models in the candidate set being 1 (Johnson & Omland, 2004).

## 3 RESULTS

### 3.1 Age pattern shape used in models

We used the quadratic shape of age for total volume and clutch size since this was the one best fitting their temporal variability, whereas we selected the log‐transformed age for egg volume for each egg order within the clutch in both two‐ and three‐egg clutches (Table 1, AICc weight of the selected models Wt_*i*_ = 0.99, 0.93, 0.96 and 0.93 respectively). For the analysis of intra‐clutch egg size profiles ($\beta_{i}$), we used the age‐logarithmic shape instead, even though the model with a continuous, linear values of age was very close and statistically equivalent (Wt_*i*_ = 0.40 and 0.43, respectively, Table 1). Since those two models were very close, we considered that the log‐transformed age shape was more realistic than a model with a linear increase of $\beta_{i}$ with age. Best fitting models (except the ones for egg volume in two‐egg clutches and models of $\beta_{i}$) showed that the selected age pattern was statistically significant (see Appendix, Table S1).

**TABLE 1:** Linear regression models for testing which age shape pattern fits better with our data for each egg parameter: total volume, clutch size, egg volume depending on egg order (separately for two‐ and three‐egg clutches) and $\beta_{i}$ during 1995–2021.

	*K*	AICc	ΔAICc	Wt	LL
Total volume					
**Age** ^**2**^	**5**	**15737.77**	**0.00**	**0.99**	**−7863.87**
Log (Age)	4	15746.35	8.58	0.01	−7869.16
Age	4	15764.36	26.60	0.00	−7878.17
Clutch size					
**Age** ^**2**^	**4**	**2711.54**	**0.00**	**0.93**	**−1351.76**
Log (Age)	3	2716.72	5.18	0.07	−1355.36
Age	3	2756.22	53.68	0.00	−1379.61
Egg volume (order) in three‐egg clutches					
**Log (Age)**	**4**	**5217.37**	**0.00**	**0.96**	**−2604.66**
Age	4	5224.08	6.71	0.03	−2608.02
Age^2^	5	5226.61	9.23	0.01	−2608.27
Egg volume (order) in two‐egg clutches					
**Log (Age)**	**4**	**2574.41**	**0.00**	**0.93**	**−1283.16**
Age	4	2580.17	5.76	0.05	−1286.04
Age^2^	5	2582.73	8.31	0.01	−1286.30
$\beta_{i}$					
**Age**	**3**	**−1301.60**	**0.00**	**0.43**	**653.82**
**Log (Age)**	**3**	**−1301.45**	**0.14**	**0.40**	**653.75**
**Age** ^**2**^	**4**	**−1299.74**	**1.86**	**0.17**	**653.91**

*Note*: Note that the age shape pattern selected here was used in the following models testing for age and its interaction with food availability (Tables 2 and 3). Models are ranked by their AICc value and selected models (models with ΔAICc < 2) are in bold.

Abbreviations: AICc, Akaike criterion for small sample sizes; *K*, number of identifiable parameters; LL, log‐likelihood of each model; Wt, weight of each model; ΔAICc, Difference in AICc value with best model.

### 3.2 Total volume, clutch size and egg order

**FIGURE 1:**
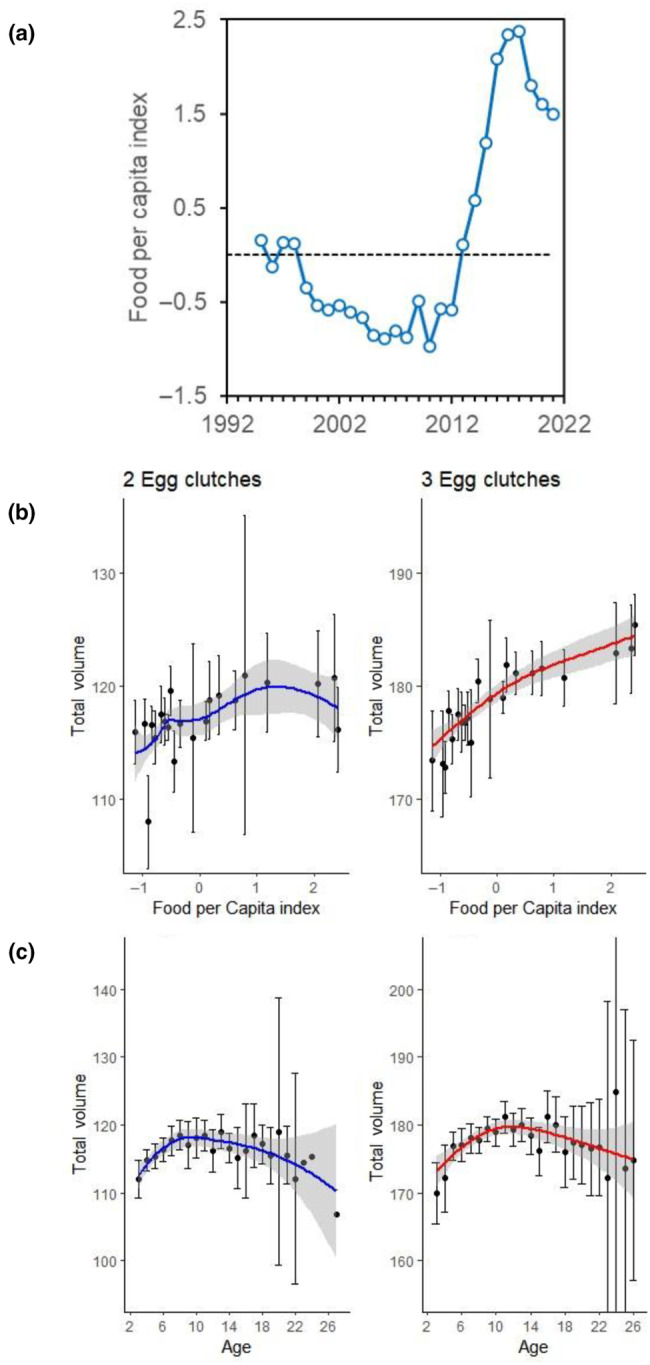
(a) Variability of food per capita index (considering intra‐ and inter‐specific competition) over the years (1995–2021). The index increased mostly due to the sharp decrease of population density of Audouin's gulls especially since 2010. Panels (b) and (c) show the relationship between total egg volume in a clutch and (a) relative index of food availability per capita and (b) age of the breeder during 1995–2021 (*N* = 2847 nests). A smoothing regression model (mean with standard error) is shown for panels (b) and (c) for assessing visually the association between variables. While age influenced similarly the total volume in clutches of two‐ and three‐eggs, food availability influenced volume much more in three‐egg than in two‐egg clutches (see Table 2).

Total volume increased with a quadratic shape of age and with the yearly availability of food per capita (Table 2 and Figure 1). While age similarly influenced the total volume of both three‐egg and two‐egg clutches following a quadratic pattern, food influenced the variability of total volume more in the former than in the latter clutches. The influence of age and food availability per capita on total volume was statistically significant (*F*_5,2068_ = 3425, *p* < .0001, see Appendix, Table S2).

**TABLE 2:** Linear models for testing the influence of food per capita and age on total volume of the clutch (considering both two‐ and three‐egg clutches), clutch size and egg volume depending on egg order (separately for two‐ and three‐egg clutches) during 1995–2021.

	*K*	AICc	ΔAICc	Wt	LL
Total volume (in two‐ and three‐egg clutches)					
**Age** ^**2**^ + Clutch*Food	**7**	**15636.77**	**0.00**	**0.78**	**−7811.36**
Age^2^ + Clutch + Food	6	15640.26	3.49	0.14	−7814.11
Clutch + Age^2^*Food	8	15642.49	5.72	0.04	−7813.21
Food + Clutch*Age^2^	8	15642.96	6.20	0.04	−7813.45
Clutch + Age^2^	5	15714.45	77.68	0.00	−7852.21
Food + Clutch	4	19743.23	4106.46	0.00	−9867.61
Clutch (null model)	3	19816.41	4179.65	0.00	−9905.20
Clutch size					
Food*Age ^2^	**7**	**2702.12**	**0.00**	**0.82**	**−1344.03**
Food + Age^2^	5	2705.27	3.15	0.17	−1347.62
Age^2^	4	2711.54	9.42	0.01	−1351.76
Food	3	3462.47	760.35	0.00	−1728.23
No effects	2	3508.01	805.89	0.00	−1752.00
Egg volume (order) in three‐egg clutches					
Log (Age) + food*order	**9**	**4871.54**	**0.00**	**0.45**	**−2426.67**
**Log (Age) + food + order**	**7**	**4871.60**	**0.06**	**0.44**	**−2428.74**
Food + Log (Age)*order	9	4875.80	4.27	0.05	−2428.80
Log (Age)* order	8	4890.94	19.41	0.00	−2437.39
Order + Log (Age)	6	4891.16	19.62	0.00	−2439.53
Food + Log (Age)	5	5203.08	331.54	0.00	−2596.51
Log (Age)	4	5217.37	345.84	0.00	−2604.66
Order + food	6	8030.99	3159.46	0.00	−4009.47
Food*order	8	8035.05	3163.51	0.00	−4009.47
Order	5	8067.50	3195.96	0.00	−4028.73
Food	4	8533.20	3661.66	0.00	−4262.58
No effects	3	8567.13	3695.59	0.00	−4280.56
Egg volume (order) in two‐egg clutches					
**Log (Age) + food + order**	**6**	**2567.49**	**0.00**	**0.34**	**−1277.66**
Food + Log (Age)*order	**7**	**2567.70**	**0.21**	**0.30**	**−1276.73**
**Food + Log (Age)**	**5**	**2569.32**	**1.83**	**0.13**	**−1279.60**
Log (Age) + food*order	7	2570.25	2.76	0.08	−1278.01
Food*order + log (Age)*order	8	2570.33	2.84	0.08	−1277.01
Order + log (Age)	5	2572.58	5.09	0.03	−1281.23
Log (Age)*order	6	2572.79	5.30	0.02	−1280.31
Log (Age)	4	2574.41	6.92	0.01	−1283.16
Food*order	6	4534.64	1967.15	0.00	−2261.27
Order + food	5	4536.58	1969.09	0.00	−2263.26
Food	4	4540.52	1973.03	0.00	−2266.24
Order	4	4557.59	1990.10	0.00	−2274.77
No effects	3	4561.46	1993.97	0.00	−2277.72

*Note*: Note that age was included considering the previous models testing the best fit for age shape pattern for each egg parameter (Table 1). Models are ranked by their AICc value and selected models (models with ΔAICc < 2) are in bold.

Abbreviations: AICc, Akaike criterion for small sample sizes; *K*, number of identifiable parameters; LL, log‐likelihood of each model; Wt, weight of each model; ΔAICc, Difference in AICc value with best model.

**FIGURE 2:**
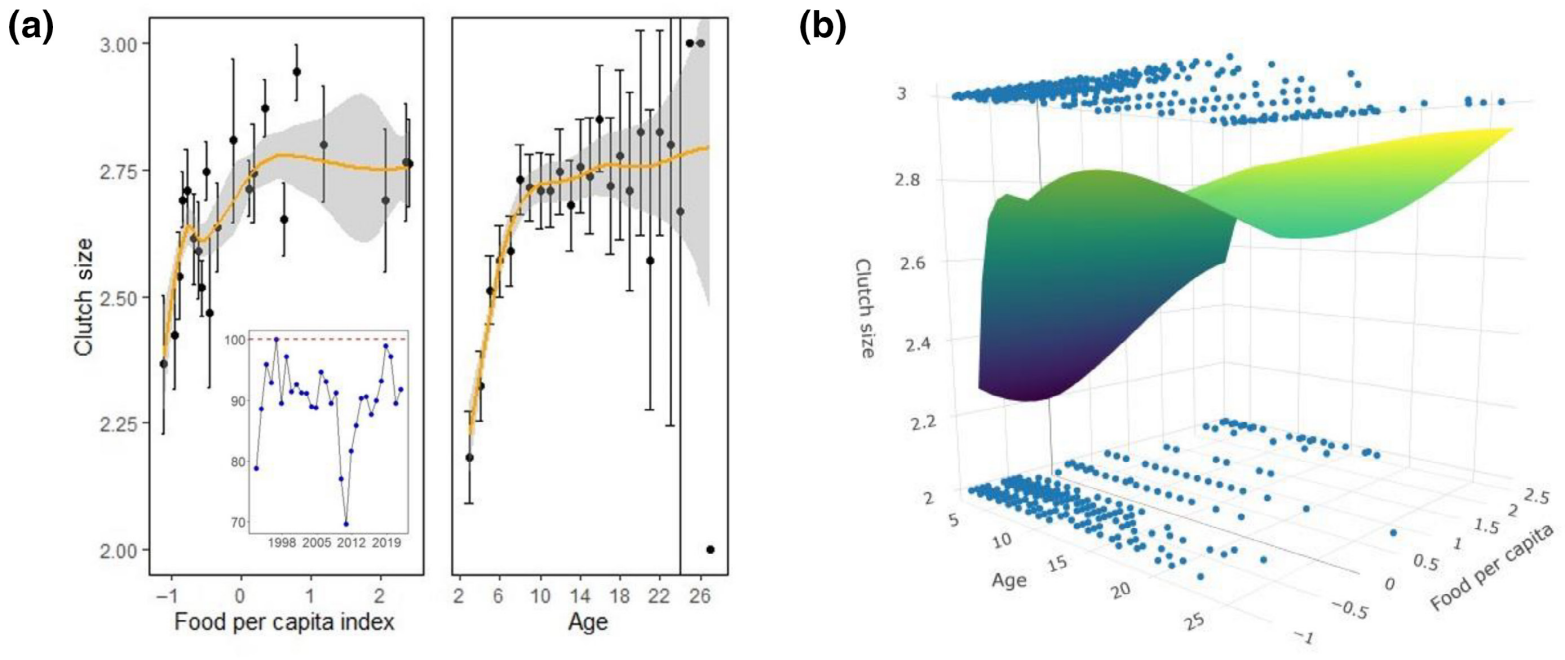
(a) Influence of food availability per capita and age on clutch size (mean and SE bars) during 1995–2021 (*N* = 2847 nests). Inset panel shows the annual variability of the percentage of three‐egg clutches over two‐egg clutches. (b) Changes in clutch size depending on the interaction between age and food availability (see selected model in Table 2). A smoothing regression model (mean with standard error) and a lowest regression surface are shown in panels (a) and (b), respectively. Note that for some ages (panel a), error bars were large (due to small sample sizes) and were not totally encompassed to ensure a proper visualization of the associations.

Results for clutch size were similar: models showed that food availability and age, the later in a quadratic shape, positively influenced the number of eggs laid by each female (Table 2 and Figure 2a). A suitable model showed that there was an interaction between food and age: we found a stronger quadratic pattern of clutch size variability in years when availability of food was lower, whereas clutch size was more linear and increased with age when food per capita was in higher supply (Figure 2b). The effects of food and age were statistically significant (*F*_5,2068_ = 40.37, *p* < .0001, see Appendix, Table S2).

**FIGURE 3:**
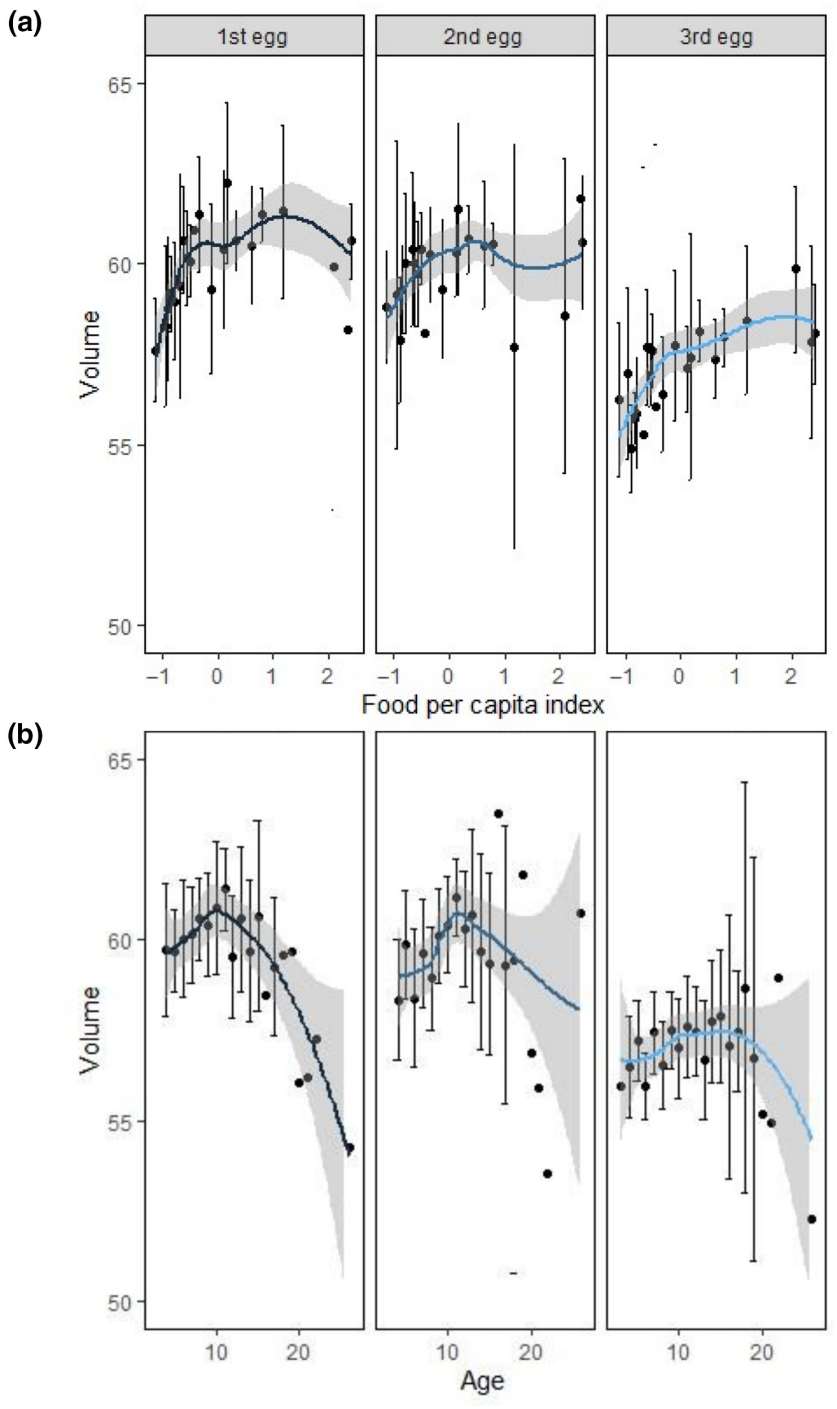
Influence of food availability per capita (a) and age (b) on egg volume in three‐egg clutches depending on egg laying order within the clutch (mean and SE bars) during 1995–2021 (*N* = 2847 nests). Models show that egg volume increased with statistically significance with higher food availability and decreased with egg order (from a‐ to c‐eggs), while it showed a quadratic influence of age (Table 2). Models also show that while the influence of age was similar for all three eggs, the influence of food was lower for b‐eggs and higher for c‐eggs compared with its influence on a‐eggs (Table 2, see also Appendix Table S2).

**FIGURE 4:**
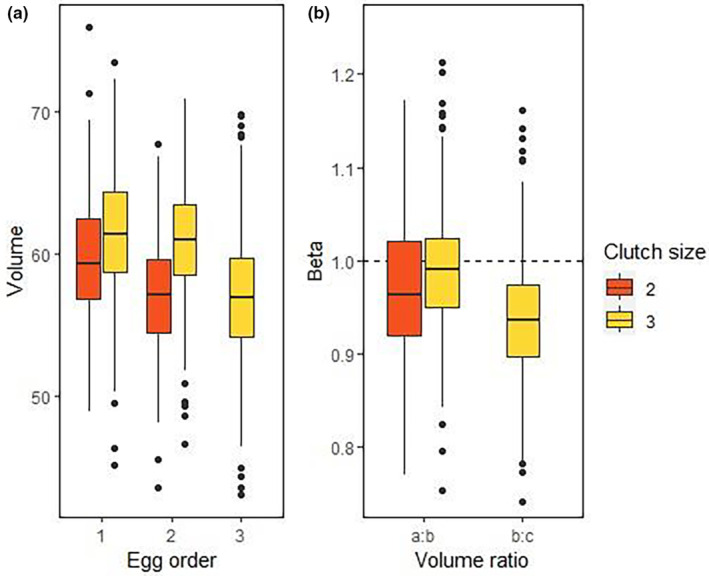
Boxplots to compare the statistical distributions of (a) the egg volume according to egg laying order and clutch size, and (b) the intra‐clutch egg volume profiles for $\beta_{i}^{{\left(a:b\right)}_{j}}$ (for each clutch size) and $\beta_{i}^{\left(b:c\right)}$ (only for three‐egg clutches), with a reference line of volume identity (β=1, i.e. the volumes are the same). Data was collected during 1995–2021 (*N* = 2847 monitored nests). Plots represent medians (bold lines), interquartile ranges (boxes), total ranges (vertical lines) and outliers (dots).

The analysis of egg size variation according to laying order in clutches of three eggs showed that a logarithmic pattern of age, food and egg order (from largest first eggs to smallest third eggs) influenced positively egg size (Figures 3 and 4a, Table 2). We found that while the influence of age was similar for all three eggs, the influence of food was lower for b‐eggs and higher for c‐eggs compared to its influence on a‐eggs (Table 2 see also Appendix, Table S2). However, a model with the additive effects of all factors (age, food, and order) was almost equivalent (Table 2). We found very similar results on egg size by laying order in clutches of two eggs: three models were statistically equivalent, although those including egg order had a large total weight (Table 2). While order and food had a statistically significant effect in egg volume for both two‐ and three‐egg clutches, the effect of age was not statistically significant (see *t*‐tests with Satterthwaite's method in Appendix, Table S2). While first and second eggs in three‐egg clutches show similar sizes and are larger than the third egg, first laid eggs in two‐egg clutches were significantly larger than second‐laid eggs (Figure 4a, see Appendix, Table S2).

### 3.3 Intra‐clutch egg size profiles

**FIGURE 5:**
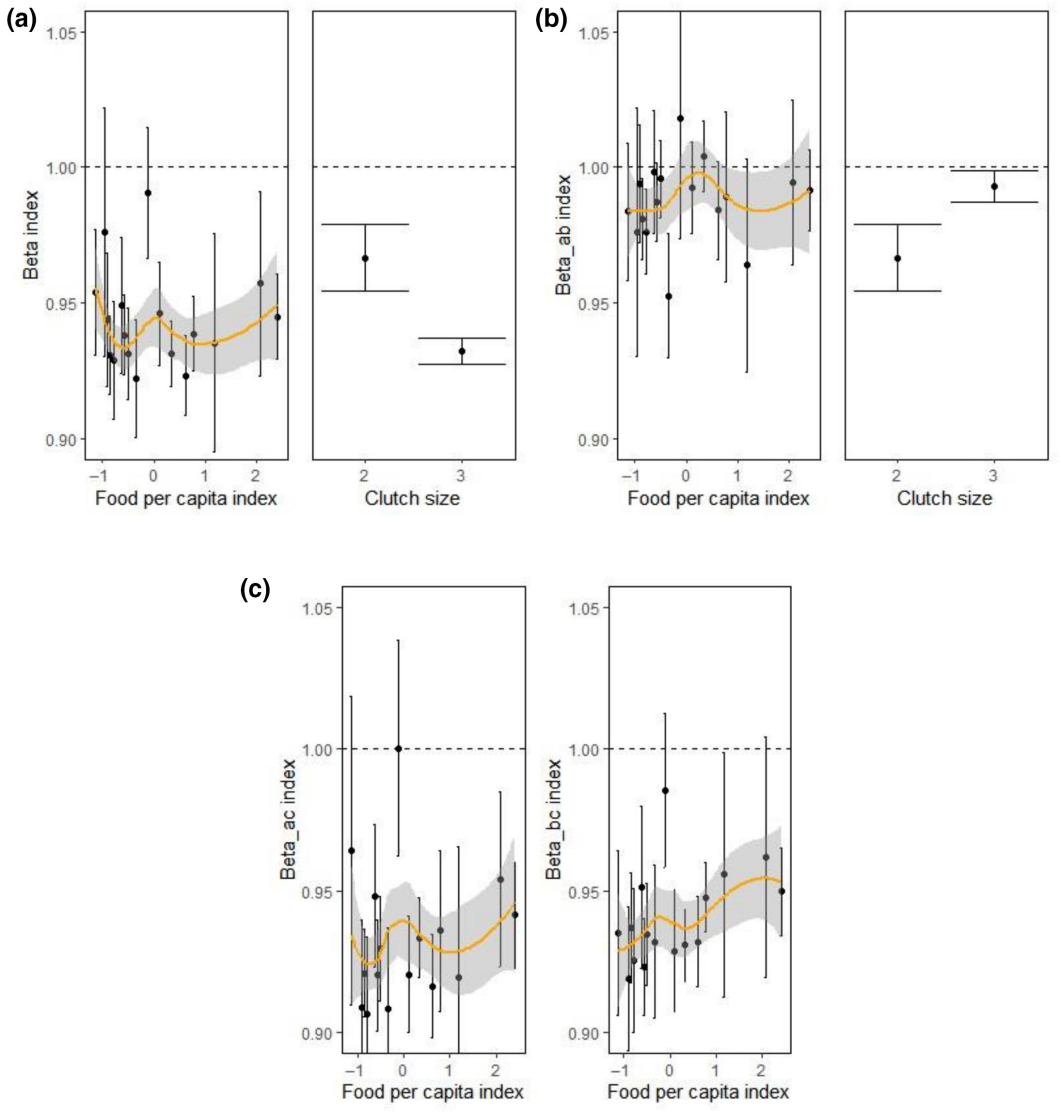
Influence of food availability per capita and clutch on egg volume profiles within the clutch, shown by the β indexes, during 1995–2021. We only considered years for which there were more than 10 clutches. The dashed line shows the reference β ratio values = 1. Food influenced all β indexes, although for $\beta_{i}^{{\left(a:b\right)}_{j}}$ and $\beta_{i}^{\left(a:c\right)}$ its effect was not statistically significant (left b and c panels, respectively). For indexes that could only be calculated for three‐egg clutches ($\beta_{i}$ and $\beta_{i}^{{\left(a:b\right)}_{j}}$; panels a and b), clutch size had a statistically significant effect. A smoothing regression model (mean with standard error) is shown in all panels for visually assessing the association between variables.

Results showed that food negatively influenced the ratio between the last egg and the precedents within the clutch *β*_*ι*_ index (Table 3, Figure 5a). This indicates that years with reduced food availability showed larger asymmetries between the last laid egg and the precedents, than years with higher food availability. As expected, the last laid egg was consistently smaller in all years and clutch sizes; that egg was smaller in relation to the precedent eggs in three‐egg clutches than in two‐egg clutches (Table 3, Figures 4a and 5a). All models testing an effect of age on *β*_*ι*_ index showed larger values of AICc (Table 3). Both food availability and clutch size in the finally selected model (Table 3) were statistically significant (*F*_2,723_ = 19.47, *p* < .0001, see Appendix, Table S2).

**TABLE 3:** Linear models for testing the influence of food per capita and age on different *β* parameters (intra‐clutch egg size profile) during 1995–2021 for two‐ and three‐egg clutches.

	*K*	AICc	ΔAICc	Wt	LL
$\beta_{i}$					
**Food + clutch**	**4**	**−1943.75**	**0.00**	**0.60**	**975.90**
Food*clutch	5	−1941.73	2.03	0.22	975.91
Clutch	3	−1941.46	2.30	0.19	973.75
No effects	2	−1909.70	34.06	0.00	956.86
Food	3	−1907.68	36.07	0.00	956.86
Food + clutch + Log (Age)	5	−1340.10	603.66	0.00	675.11
Food + clutch*Log (Age)	6	−1339.73	604.03	0.00	675.95
Clutch + Log (Age)	4	−1338.44	605.31	0.00	673.26
Log (Age)	3	−1301.60	642.16	0.00	653.82
Food + Log (Age)	4	−1299.93	643.83	0.00	654.00
Food*Log (Age)	5	−1298.14	645.62	0.00	654.13
$\beta_{i}^{{\left(a:b\right)}_{j}}$					
**Clutch**	**3**	**−1747.65**	**0.00**	**0.63**	**876.84**
**Food + clutch**	**4**	**−1745.91**	**1.74**	**0.26**	**876.98**
Clutch*food	5	−1744.18	3.47	0.11	877.13
No effects	2	−1733.85	13.80	0.00	868.93
Food	3	−1732.54	15.11	0.00	869.29
Clutch + Log (Age)	4	−1360.14	387.52	0.00	684.11
Food + clutch + Log (Age)	5	−1358.45	389.20	0.00	684.29
Food + clutch*Log (Age)	6	−1357.73	389.92	0.00	684.95
Log (Age)	3	−1356.54	391.11	0.00	681.30
Food + Log (Age)	4	−1354.52	393.13	0.00	681.30
Food*Log (Age)	5	−1352.59	395.06	0.00	681.35
$\beta_{i}^{\left(a:c\right)}$					
**Food**	**3**	**−1331.28**	**0.00**	**0.50**	**667.65**
**No effects**	**2**	**−1331.27**	**0.01**	**0.50**	**668.66**
Food + Log (Age)	4	−992.86	338.41	0	500.48
Log (Age)	3	−991.80	339.47	0	498.93
Food*Log (Age)	5	−990.99	340.28	0	500.57
$\beta_{i}^{\left(b:c\right)}$					
**Food**	**3**	**−1560.56**	**0.00**	**0.88**	**783.30**
No effects	2	−1556.58	3.98	0.12	780.30
Food + Log (Age)	4	−1011.98	548.58	0	510.04
Food*Log (Age)	5	−1011.03	549.53	0	510.59
Log (Age)	3	−1009.03	551.53	0	507.55

*Note*: Models are ranked by their AICc value, and selected models (models with ΔAICc < 2) are in bold. $\beta_{i}$ last egg asymmetry; $\beta_{i}^{{\left(a:b\right)}_{j}}$ first and second egg asymmetry; $\beta_{i}^{\left(a:c\right)}$ first and third egg asymmetry; $\beta_{i}^{\left(b:c\right)}$ second and third egg asymmetry.

Abbreviations: AICc, Akaike Criterion for small sample sizes; *K*, number of identifiable parameters; LL, log‐likelihood of each model; Wt, weight of each model; ΔAICc, Difference in AICc value with best model.

The selected model on the ratio between the a‐ and b‐egg volumes $\beta_{i}^{{\left(a:b\right)}_{j}}$ showed that the ratio was influenced by clutch size and was larger in two‐egg clutches (Table 3, Figures 4a and 5b). The effect of clutch size was statistically significant (*F*_1,724_ = 15.95, *p* < .0001, see Appendix, Table S2). A second suitable model showed that increased food availability might decrease that ratio (i.e. a‐ and b‐eggs had more similar values; Table 3, Figure 5b). Finally, none of the selected models showed an effect of age on $\beta_{i}^{\left(a:b\right)}$ (Table 3).

Analyses comparing the ratio between the c‐egg volume and the a‐ and the b‐egg volume ($\beta_{i}^{a:c}$ and $\beta_{i}^{b:c}$) in three‐egg clutches showed that age did not play a significant role (Table 3). Even though the best models showed a positive effect of food availability on $\beta_{i}^{a:c}$ and $\beta_{i}^{b:c}$ (Table 3, Figure 5c), that effect was only statistically significant for the latter (*F*_1,575_ = 2.01, *p* = .157 and *F*_1,575_ = 6.01, *p* < .015 respectively, see Appendix, Table S2).

Finally, the egg volume profiles in two‐egg clutches (a:b, i.e. $\beta_{i}^{{\left(a:b\right)}_{2}}$) was more similar to a b:c profile ($\beta_{i}^{b:c}$) than to an a:b profile ($\beta_{i}^{{\left(a:b\right)}_{3}}$) in three‐egg clutches (Figure 4a,b, see Appendix, Table S3). The statistical differences were less marked between the a:b profile in two‐egg clutches and the b:c profile than between the a:b profiles in two‐ and three‐egg clutches (ANOVA *F*_1,724_ = 23.49, *p* = 1.53e‐06; *F*_1,1729_ = 254.90, *p* = 2.00e‐16 respectively; Figure 4b). This was confirmed by the more similar volumes between the a‐ and b‐eggs in three‐egg clutches than in two‐egg clutches (*F*_1,1301_ = 18.01, *p* < 2.36e‐05; Figure 4b).

## 4 DISCUSSION

How female birds invest in the volume and count of their eggs under variable environments remains poorly understood. There are currently many constraints to collecting long‐term data on stochastic food conditions and the range of ages for breeders (Christians, 2002; Monaghan & Nager, 1997). Due to the difficulties involved in obtaining this data, those two factors are commonly considered independently (but see Hernández et al., 2015; Oro et al., 2014; Pardo et al., 2012; Tompkins & Anderson, 2021).

### 4.1 Food availability and breeding investment

Our investigation into the intricate interplay between environmental factors, age, and reproductive strategies in Audouin's gulls revealed compelling insights. Central to our study was the profound influence of food availability on various facets of breeding investment. Across all parameters under scrutiny, per capita food supply variations yielded significant effects. Food influenced the amount of energy allocated for producing eggs (their size and number) and the way of allocating those energies in each egg (i.e. asymmetries within the clutch; Table 4). This underscores the pivotal role of environmental conditions in shaping breeding investment strategies, a phenomenon well supported by earlier studies across different avian species. These studies involved other gull species (Bolton, 1991; Oro, 1996; Pierotti, 1982; Pons, 1992; Sydeman & Emslie, 1992), other seabirds (Morris & Chardine, 1995; Oro, 1999), raptors (Valkama et al., 2002; Wiebe & Bortolotti, 1996), ducks (Flint et al., 1992) and rails (Sanchez‐Lafuente, 2004), among others. The pivotal role of food availability in influencing reproductive decisions aligns with the fundamental tenets of life history theory, where organisms allocate resources optimally to enhance fitness in varying ecological contexts (Stearns, 1976).

**TABLE 4:** Summary of results on the effects of food and age (and clutch size in some cases) on laying parameters.

Parameter	Effects		
	Food	Age	Interactions
Total egg volume	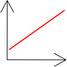	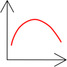	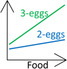
Clutch size	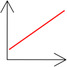	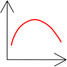	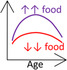
Egg volume within clutches	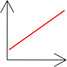	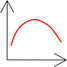	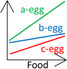
Intra‐clutch egg size profiles			
*β*_*ι*_	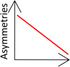	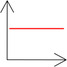	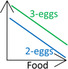
$\beta_{i}^{{\left(a:b\right)}_{j}}$	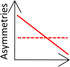	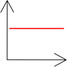	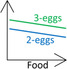
$\beta_{i}^{a:c}$	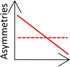	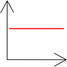	NA
$\beta_{i}^{b:c}$	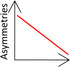	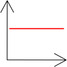	NA

*Note*: We tested both individual effects and their interactions in all models (see Tables 1 and 2). Dashed lines show results from statistically equivalent models than solid lines.$\;\beta_{i}$ last egg asymmetry; $\beta_{i}^{{\left(a:b\right)}_{j}}$ first and second egg asymmetry; $\beta_{i}^{\left(a:c\right)}$ first and third egg asymmetry; $\beta_{i}^{\left(b:c\right)}$ second and third egg asymmetry.

Females may also allocate energy differently in each egg within the clutch, as asymmetries would influence the features of sibling competition (Parker & Begon, 1986). This is particularly relevant in species where differences in egg size may lead to differences in hatchling viability (Ramírez et al., 2015; Saino et al., 2010; Sydeman & Emslie, 1992). We found that the influence of food availability on volume occurred for all eggs in the clutch (see also Williams & Cooch, 1996). This did not agree with what was predicted by an energetic model trying to explain how clutches are formed in bet‐hedging gulls (Ruiz et al., 2000). This model predicted that significant increases of the durations of the rapid yolk development (RYD) periods of second and third eggs would determine its constancy in volume independently of food conditions. Instead, our results agree with previous studies finding that last laid eggs within clutches were smaller, especially when there was a shortage of food (Kilpi, 1995; Kilpi et al., 1996; Robertson, 1995). This suggests that the characteristic smaller size of last laid eggs in clutches of gulls is an intrinsic consequence of the clutch formation mechanism and indeed does depend on food conditions. We actually found that profiles in two‐egg clutches corresponded to a first (*a*‐ or *b*‐) plus a last laid egg (*c‐*), rather than to two first laid (*a*‐ and *b*‐) eggs (Figure 4a). In other words, a two‐egg clutch produces a larger first egg and is followed up by a significantly smaller egg. This is contrary to the idea that last laid eggs become smaller as food availability is depleted until the clutch size switches from three to two eggs (Kilpi et al., 1996; Winkler, 1985).

### 4.2 Age and reproductive strategies

There is also evidence that, apart from food, age influences breeding investment and performance in birds, especially in long‐lived species (Aubry et al., 2009; Forslund & Pärt, 1995; Hernández et al., 2015; Laaksonen et al., 2002; Mills, 1979). Intriguingly, age in Audouin's gull influenced only the parameters of egg and clutch sizes, whereas the asymmetries among the eggs remained constant for all age classes (Table 4). A few empirical studies found that age did not influence egg size profiles within the clutch (Catry & Furness, 1997; Polito et al., 2010). Owing that age is a proxy of experience and performance, the consistency in intra‐clutch asymmetries for all age classes suggests that decreasing egg size with laying order in many birds may constitute a process occurring at two scales. From a life history perspective, this suggests an adaptive strategy of parental favouritism towards early hatching offspring with larger reproductive value. From an ecological perspective, a result from nutritional constraints on laying effort (Ramírez et al., 2011; Wiebe & Bortolotti, 1996; Winkler, 1985). The quadratic pattern of age‐related effects on egg and clutch sizes suggests differential performance between younger and older breeders. This indicates that younger and older breeders performed worst, the former likely resulting from inexperience and the latter from senescence (Christians, 2002; Froy et al., 2017; Oro et al., 2014; Pardo et al., 2012). These observations highlight the complex interaction between intrinsic factors like age and extrinsic factors like food availability in shaping reproductive outcomes.

### 4.3 Interaction between food and age on breeding investment

Our study extends this understanding by demonstrating the dynamic interplay between age and food availability, further emphasizing the adaptive flexibility of breeding strategies in response to environmental conditions. For instance, navigating the complexities within two‐egg clutches introduced an additional layer of intricacy. These clutches were predominantly associated with younger or older breeders during years of large food availability. This can be explained because those breeders in those years should be affected more by age and other individual constraints (e.g. genotypic quality, mate efficiency), than by food limitations. Thus, such females produced a sub‐optimal clutch size as an outcome of their intrinsically poor performance because they most likely relied on stored reserves (capital tactic, sensu Jönsson, 1997) than their three‐egg clutch counterparts (Ruiz, González‐Solıs, et al., 1998; Ruiz, Jover, & Pedrocchi, 1998). In fact, clutch size varied less between age classes as food was in shorter supply, which suggests that under poor environmental conditions, only high‐quality individuals of the younger and older age classes bred, whereas low‐quality individual would skipping breeding (Bradley et al., 2000; see also Oro et al., 2014; Reed et al., 2015). The contrasting strategies of younger, inexperienced breeders, and older, senescent individuals, emphasize the diverse approaches adopted by different age classes to navigate stochastic environmental challenges. Our study echoes findings from diverse avian species, highlighting the multifaceted ways in which age and food impacts reproductive decisions (Christians, 2002; Reid, 1987a; Winkler, 1985).

### 4.4 Exploring bet‐hedging strategies

Our results indeed align closely with the principles of bet‐hedging—a strategy enabling organisms to mitigate risks posed by unpredictable environments. Audouin's gulls show a reduced investment in reproduction in adverse conditions, as even a small decrease in adult survival can significantly lower the number of future breeding seasons and overall reproductive success (Curio, 1983). Experimental studies supplementing food for breeders show that gulls refrain from laying larger clutches than the modal size of three eggs (Bolton et al., 1992, 1993; Nager et al., 2000). This bet‐hedging strategy for an organism with high chances of numerous breeding attempts over its lifetime is supported by the potential stochasticity on food conditions within the breeding season. After hatching, those conditions may vary and this may decouple the advantages of having laid more and larger eggs (Bolton, 1991; Bolton et al., 1992; see review in Christians, 2002). Even though we could not record the number of chicks raised by each monitored bird, results suggest that the size and number of eggs produced by Audouin's gulls produce the optimal number of chicks that may be raised in a breeding season without incurring fitness survival costs, given the stochastic nature of food availability (Drent & Daan, 1980; Nager et al., 2000). This adaptive strategy enables individuals to diversify offspring traits, increasing the likelihood of survival in unpredictable, stochastic conditions.

### 4.5 Acknowledging limitations and uncertainties

Turning our attention to the limitations of our study, we recognize potential sources of uncertainty. First, we did not consider clutches of one and four eggs. Furthermore, one notable assumption was the exclusive reliance on discards as the primary food source for Audouin's gulls. While this assumption enabled us to streamline the investigation, it may oversimplify the complexity of the gulls' foraging ecology (Oro et al., 1997). Individuals likely engage with multiple food sources, other than just these discards, potentially impacting their reproductive investment strategies. Additionally, unaccounted for variables like breeding site quality, phenology and genetic diversity might interact with the factors studied, influencing the complex relationship between food availability, age, and reproductive investment. We underscore the need for cautious interpretation and encourage future studies to consider a broader spectrum of ecological factors on bet‐hedging strategies.

### 4.6 Conclusions, implications and future directions

Our study provides a comprehensive exploration of the intricate relationship between food availability, age and reproductive strategies in Audouin's gulls. Through an in‐depth analysis, we unveil the complexities of resource allocation, egg and clutch size dynamics and intra‐clutch asymmetries. The overarching theme of bet‐hedging emerges as a unifying principle governing breeding decisions in unpredictable conditions. While our current analysis is empirical, further exploration into the theoretical foundations of bet‐hedging and avian evolutionary biology would be valuable (Starrfelt & Kokko, 2012).

## AUTHOR CONTRIBUTIONS

**Daniel Oro:** Conceptualization (lead); formal analysis (equal); investigation (lead); supervision (lead); writing – original draft (lead); writing – review and editing (equal). **Cassidy Waldrep:** Data curation (supporting); formal analysis (equal); writing – review and editing (equal). **Albert Bertolero:** Data curation (supporting). **Meritxell Genovart:** Conceptualization (supporting); supervision (supporting); writing – review and editing (supporting).

## Supporting information


Table S1

## Data Availability

Data on egg and clutch size for each nest are available at CSIC open repository https://doi.org/10.20350/digitalCSIC/14795.
