# Supplementary material for: Drivers of resource allocation for breeding under variable environments in a bet hedger
Source: Ecol Evol. 2023 Sep 6;13(9):e10485. doi: 10.1002/ece3.10485 (PMC10483097; doi:10.1002/ece3.10485)
Supplement: Supplementary file 1 — Table S1 [file ECE3-13-e10485-s001.docx]

Supplementary material

Table S1. Summary statistics (*F*- and *t*-statistics for GLM and GLMM respectively, p-values) and estimates (and error terms) for the fixed effects in top models for each linear regression analysis to test which age shape pattern best fitted the data for each egg parameter (see models in Table 1). For GLMM models, *t*-tests used Satterthwaite's method for calculating the statistical significance. For GLM models, *F*-statistic was also provided together with its degrees of freedom. Notation is SE: standard error of the estimates; df: degrees of freedom; p: level of statistical significance (0 ‘***’, 0.001 ‘**’, 0.01 ‘*’, 0.05 ‘.’, 0.1 ‘ ’, 1).

| Total volume for age shape (Table 1) |  |  |  |  |  |
| --- | --- | --- | --- | --- | --- |
|  | Estimate | SE | df | *t*-statistic | *p* |
| Intercept | 1.39e+02 | 3.05e+01 | 1.00e+00 | 4.585 | 0.136 |
| Age | 1.42e+00 | 2.13e-01 | 2.07e+03 | 6.686 | 2.9e-11 *** |
| Age^2^ | -5.45e-02 | 9.03e-03 | 2.07e+03 | -6.040 | 1.8e-09 *** |
| Clutch size for age shape (Table 1) | Estimate | SE | df | *t*-statistic | *p* |
| Intercept | 2.079 | 0.046 |  | 44.595 | < 2e-16 *** |
| Age | 0.091 | 0.009 |  | 10.123 | < 2e-16 *** |
| Age^2^ | -0.002 | 0.000 |  | -7.508 | 8.9e-14 *** |
| *F*-statistic | 92.63 |  | 2, 2071 |  | < 2e-16 *** |
| Egg order for 3-egg clutches (Table 1) | Estimate | SE | df | *t*-statistic | *p* |
| Intercept | 56.539 | 1.011 | 367.446 | 55.882 | <2e-16 *** |
| Log(Age) | 0.984 | 0.450 | 368.362 | 2.184 | 0.0296 * |
| Egg order for 2-egg clutches (Table 1) | Estimate | SE | df | *t*-statistic | *p* |
| Intercept | 59.479 | 1.173 | 241.608 | 50.686 | <2e-16 *** |
| Log(Age) | 0.689 | 0.523 | 241.191 | 1.317 | 0.189 |
| $\beta_{i}$ | Estimate | SE | df | *t*-statistic | *p* |
| Intercept | 0.925 | 0.014 |  | 64.622 | <2e-16 *** |
| Log(Age) | 0.005 | 0.006 |  | 0.913 | 0.362 |
| *F*-statistic | 0.833 |  | 1, 497 |  | 0.362 |
|  |  |  |  |  |  |
|  |  |  |  |  |  |

Table S2. Summary statistics (*F*- and *t*-statistics for GLM and GLMM respectively, p-values) and estimates (and error terms) for the fixed effects in top models for each linear regression analysis for each egg parameter (see models in Table 2). For GLMM models, *t*-tests used Satterthwaite's method for calculating the statistical significance. For GLM models, *F*-statistic was also provided together with its degrees of freedom. Notation is SE: standard error of the estimates; df: degrees of freedom; p: level of statistical significance (0 ‘***’, 0.001 ‘**’, 0.01 ‘*’, 0.05 ‘.’, 0.1 ‘ ’, 1). For egg volume and order in 3-egg clutches, we used the model with all additive factors (see Table 2).

| Total volume (Table 2) |  |  |  |  |  |
| --- | --- | --- | --- | --- | --- |
|  | Estimate | SE | df | *t*-statistic | *p* |
| Intercept | 109.918 | 1.064 |  | 103.275 | < 2e-16*** |
| Food | 1.271 | 0.462 |  | 2.748 | 0.00605** |
| Clutch | 60.923 | 0.512 |  | 118.822 | < 2e-16*** |
| Age | 1.402 | 0.209 |  | 6.701 | 2.66e-11*** |
| Age^2^ | -0.054 | 0.008 |  | -6.138 | 9.99e-10*** |
| Food * clutch | 1.284 | 0.548 |  | 2.344 | 0.01917* |
| *F*-statistic | 3425 |  | 5, 2068 |  | < 2e-16*** |
| Clutch size (Table 2) | Estimate | SE | df | *t*-statistic | *p* |
| Intercept | 2.098 | 0.047 |  | 44.646 | <2e-16*** |
| Food | 0.109 | 0.044 |  | 2.449 | 0.014 * |
| Age | 0.089 | 0.009 |  | 9.929 | <2e-16 *** |
| Age^2^ | -0.002 | 0.000 |  | -7.420 | 1.7e-13 *** |
| Food*Age | -0.014 | 0.008 |  | -1.652 | 0.098 . |
| Food*Age^2^ | 0.001 | 0.000 |  | 1.570 | 0.116 |
| *F*-statistic | 40.37 |  | 5, 2068 |  | <2e-16*** |
| Egg order for 3-egg clutches (Table 2) | Estimate | SE | df | *t*-statistic | *p* |
| Intercept | 59.457 | 0.961 | 406.685 | 61.814 | <2e-16*** |
| Order (2) | -0.537 | 0.254 | 490.836 | -2.115 | 0.0349 * |
| Order (3) | -4.569 | 0.240 | 577.847 | -18.966 | <2e-16*** |
| Food | 0.844 | 0.173 | 398.345 | 4.878 | 1.55e-06 *** |
| Log(Age) | 0.826 | 0.421 | 385.951 | 1.961 | 0.050 . |
| Egg order for 2-egg clutches (Table 2) | Estimate | SE | df | *t*-statistic | *p* |
| Intercept | 60.057 | 1.666 | 244.917 | 51.470 | <2e-16*** |
| Order (2) | -0.507 | 0.225 | 239.289 | -2.249 | 0.0254 * |
| Food | 0.637 | 0.219 | 237.832 | 2.908 | 0.0039 ** |
| Log(Age) | 0.568 | 0.517 | 239.888 | 1.098 | 0.273 |
| $\beta_{i}$ | Estimate | SE | df | *t*-statistic | *p* |
| Intercept | 0.970 | 0.005 |  | 175.242 | <2e-16*** |
| Food | 0.005 | 0.002 |  | 2.077 | 0.0382 * |
| Clutch | -0.038 | 0.006 |  | -6.241 | 7.41e-10*** |
| *F*-statistic | 19.47 |  | 2,723 |  | 5.783e-09 |
| $\beta_{i}^{\left( a:b \right)_{j}}$ | Estimate | SE | df | *t*-statistic | *p* |
| Intercept | 0.966 | 0.005 |  | 162.930 | <2e-16*** |
| Clutch | 0.026 | 0.006 |  | 3.993 | 7.18e-05 *** |
| *F*-statistic | 15.95 |  | 1,724 |  | 7.18e-05 *** |
| $\beta_{i}^{a:c}$ | Estimate | SE | df | *t*-statistic | *p* |
| Intercept | 0.929 | 0.003 |  | 293.173 | <2e-16*** |
| Food | 0.004 | 0.003 |  | 1.417 | 0.157 |
| *F*-statistic | 2.008 |  | 1,575 |  | 0.157 |
| $\beta_{i}^{b:c}$ | Estimate | SE | df | *t*-statistic | *p* |
| Intercept | 0.937 | 0.002 |  | 360.538 | <2e-16*** |
| Food | 0.006 | 0.002 |  | 2.452 | 0.0145 * |
| *F*-statistic | 6.013 |  | 1,575 |  | 0.0145 |

Table S3. Descriptive statistics of egg volume (in cc) for each egg order and clutch size (see Fig. 4 in the main text). We show the mean and the standard deviation (SD) values for each egg in the clutch and the t-test for testing the difference in volumes; a- and b- represent the first and second egg in the clutch; N = sample sizes; df = degrees of freedom.

| Egg order | Clutch size | mean | SD | N | t-test (df) | p-value |
| --- | --- | --- | --- | --- | --- | --- |
| a- | 2 | 59.48 | 4.37 | 248 |  |  |
|  | 3 | 61.42 | 4.16 | 482 | 0.054 (976) | 0.957 |
| b- | 2 | 57.03 | 4.27 | 248 |  |  |
|  | 3 | 61.02 | 4.06 | 482 | 0.558 (678) | 0.557 |
|  |  |  |  |  |  |  |
|  |  |  |  |  |  |  |
